# Effects of the PIWI/MID domain of Argonaute protein on the association of miRNAi's seed base with the target

**DOI:** 10.1261/rna.069328.118

**Published:** 2019-05

**Authors:** Zhen Wang, Yanli Wang, Taigang Liu, Yujie Wang, Wenbing Zhang

**Affiliations:** Department of Physics, Wuhan University, Wuhan, Hubei 430072, P.R. China

**Keywords:** RNA interference, Argonaute protein, seed, molecular dynamic simulation

## Abstract

The small interfering RNAs (siRNA) or microRNAs (miRNA) incorporated into the RNA-induced silencing complex with the Argonaute (Ago) protein associates with target mRNAs through base-pairing, which leads to the cleavage or knockdown of the target mRNA. The seed region of the s(m)iRNA is crucial for target recognition. In this work, a molecular dynamic simulation was utilized to study the thermodynamics and kinetic properties of the third seed base binding to the target in the presence of the PIWI/MID domain of Ago. The results showed that in the presence of the PIWI/MID domain, the entropy and enthalpy changes for the association of the seed base with the target were smaller than those in the absence of protein. The binding affinity was increased due to the reduced entropy penalty, which resulted from the preorganization of the seed base into the A-helix form. In the presence of the protein, the association barrier resulting from the unfavorable entropy loss and the dissociation barrier coming from the destruction of hydrogen bonding and base-stacking interactions were lower than those in the absence of the protein. These results indicate that the seed region is crucial for fast recognition and association with the correct target.

## INTRODUCTION

**FIGURE 1.**
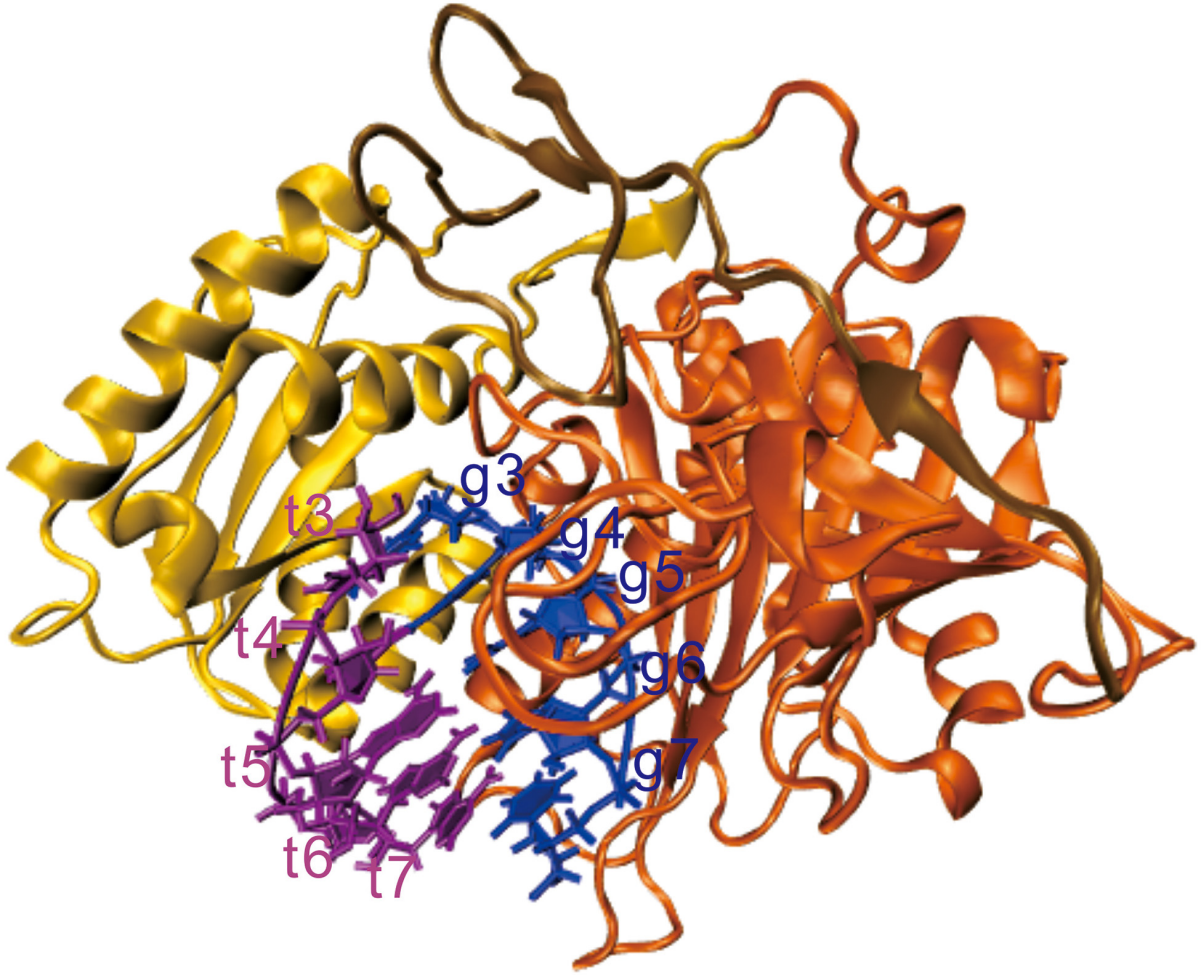
The structure of the model system (PDB ID: 4W5O). The MID domain (yellow), the PIWI domain (orange), the L2 linker (tan), the seed region of miRNA (blue), and the target RNA (purple).

RNA interference (RNAi) is induced by double-stranded RNA (dsRNA) and results in gene silencing of the target RNA. There are two types of RNAi: One is known as small interfering RNAs (siRNA), which is triggered by dsRNA helices having been introduced exogenously into cells; the other one is microRNA (miRNA), which is produced endogenously from small noncoding RNAs (Hammond et al. 2000; Zamore et al. 2000; Liu et al. 2004; Wang et al. 2008b). One strand of the s(m)iRNA duplex is loaded into the Argonaute (Ago) protein, forming the RNA-induced silencing complex (RISC) (Bartel 2004; Song et al. 2004; Tang 2005; Peters and Meister 2007; Hutvagner and Simard 2008; Kawamata and Tomari 2010; Wilson and Doudna 2013); and then the s(m)iRNA within the RISC associates with target mRNAs through base-pairing (Hutvágner and Zamore 2002; Martinez et al. 2002; Saito and Sætrom 2010), resulting in the cleavage or translation repression and destabilization of the target mRNA (Doench and Sharp 2004; Gregory et al. 2005). An RNA silence mechanism, such as the interaction and affinity between miRNA and mRNA in the presence of Ago, has attracted considerable interest and has been studied extensively in recent years (Martinez and Tuschl 2004; Vella et al. 2004; van den Berg et al. 2008; Wang et al. 2010; Chen and Zhang 2012; Dieterich and Stadler 2013; Gan and Gunsalus 2013; Khorshid et al. 2013; Schuck et al. 2013). Biochemical and bioinformatic studies indicated that Ago proteins divide s(m)iRNAs into five distinct domains, and the seed region that resides at the 5′-end of miRNAs and spans from nucleotide positions 2 to 7 was crucial for target recognition (Lewis et al. 2005; Bartel 2009; Pasquinelli 2012; Gorski et al. 2017). The crystal structures (see Fig. 1) of the RNA–protein complex show that the seed region of the guide strand is settled by its phosphodiester backbone to the PIWI/MID domain of Argonaute protein, and the seed region in an A-form helix is important for target recognition (Ma et al. 2005; Parker et al. 2005; Wang et al. 2008a). It has been found that the affinity of the seed-target interaction was greatly increased upon the association of the AfPiwi protein, a model of the PIWI/MID domain of the Argonaute protein (Parker et al. 2009). Single molecule experiments showed that incorporation of the guide RNA into RNP greatly increased the association rate constant (Chandradoss et al. 2015; Jo et al. 2015; Salomon et al. 2015). The RNP finds its targets less efficiently and binds to them less stably without a matched seed sequence.

Since its discovery, many methods have been developed to predict miRNA and its targets through thermodynamic properties (Enright et al. 2003; Lewis et al. 2003; Krek et al. 2005; Hsu et al. 2006; Krüger and Rehmsmeier 2006; Maragkakis et al. 2009; Peterson et al. 2014). However, due to lack of thermodynamic parameters of RNA with the protein, the seed's effects could not efficiently incorporate the methods, and the accuracies are limited. The methods could improve their performance by using more accurate thermodynamic parameters of RNA with the protein. Molecular dynamic simulation as a powerful tool has been used to study the thermodynamic and kinetic properties of RNA (Várnai et al. 2004; Zhang et al. 2008, 2011; Gong et al. 2011; Nguyen et al. 2017). Recently, the simulation has been used to obtain the quantitative thermodynamic and kinetic parameters of the terminal base pair upon opening-closing transition (Wang et al. 2016, 2018). In this paper, we obtained the thermodynamic and kinetic properties of the third seed base binding to the target in the presence of the Argonaute protein through simulating the association-dissociation switch of the base pair. The results showed that the binding affinity was increased due to the reduced entropy penalty, which resulted from the preorganization of the seed base into the A-helix form. The association barrier resulting from the unfavorable entropy loss and the dissociation barrier coming from the disruption of hydrogen bonding and base-stacking interactions in the presence were lower than those in the absence of the protein, leading to the increase of the rate constant with which it binds to the target.

## RESULTS AND DISCUSSION

### The nucleotide of the miRNA in the seed region is preorganized

**FIGURE 2.**
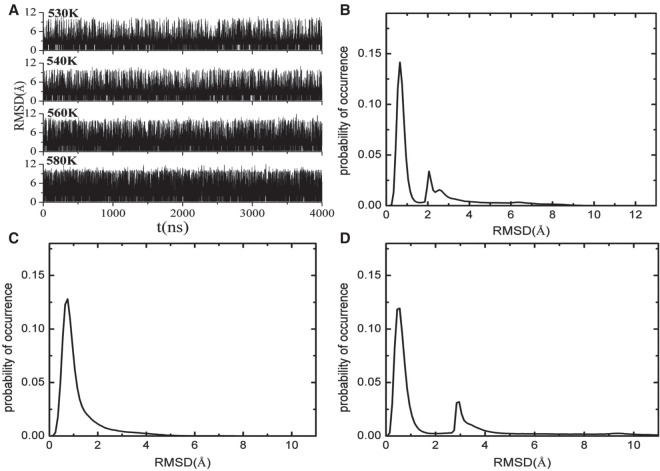
(*A*) The RMSD of guide and target bases during the whole simulation time at the temperatures of 530, 540, 560, and 580 K. (*B*) The distribution of RMSD for the seed and target bases at 540 K. (*C*) The distribution of RMSD for the seed base at 540 K. (*D*) The distribution of RMSD for target base at 540 K.

**FIGURE 3.**
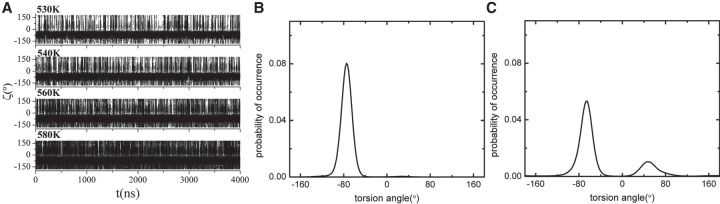
(*A*) The torsion angle of the target base during the whole simulation time at temperatures of 530, 540, 560, and 580 K. (*B*) The distribution of the torsion angle of the seed base at 540 K. (*C*) The distribution of the torsion angle of the target base at 540 K.

**FIGURE 4.**
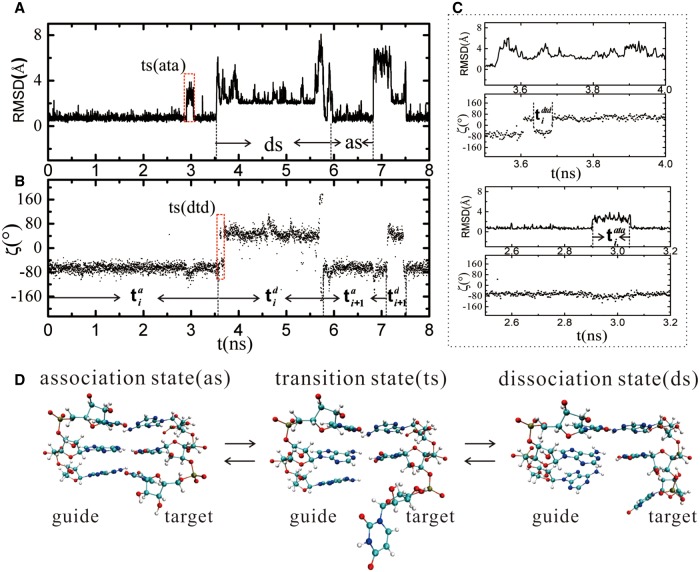
(*A*) The RMSD of the seed and target bases during the simulation times of 0–8 nsec at 540 K. (*B*) The torsion angles ζ of the target base for the simulation times of 0 –8 nsec at 540 K. (*C*) The RMSD and torsion angles ζ near the transition states. (*D*) The atomic structures of the association, dissociation, and transition states (ata and dtd).

As has been shown, the terminal base pair would go through the open–close transition through a transition state when there is no protein (Wang et al. 2016, 2018). The open state, closed state, and transition state can be classified from the root mean square deviation (RMSD) of the two terminal nucleotides relative to their starting structures (see Figs. 2A, 4A), the backbone torsion angles ζ (see Figs. 3A, 4B), and the dihedral angle of the four atoms C3′(n)-O3′(n)-P(n + 1)-O5′(n + 1) (Hershkovitz et al. 2003), where n represents the nth nucleotide in a polynucleotide chain.

Closed state: The conformations are considered as a closed state when the RMSD of the terminal bases is ∼0.7 Å and the torsion angle ζ is between −50° and −100° (see Fig. 4A,B). At this state, the two terminal bases are only vibrating at their starting locations, and the base pair and base stacking are intact.

Open state: The conformations are classified as open state when the RMSD is larger than 2 Å and the torsion angle ζ is between 50° and 100° (see Fig. 4A,B). At this state, the two bases deviated away from the starting positions and the terminal bases flipped into the solvent.

Transition state: The conformations are denoted as transition state when the RMSD of terminal bases is larger than 2 Å, but the torsion angle ζ remains between −50° and −100° (see Fig. 4C). In this state, the torsion angle was still in the closed state region while the bases were flipped out into the solvent (see Fig. 4D). The transition states can be divided into ctc and oto (Dokholyan et al. 2000; Zhang et al. 2006); ctc is defined as the transition state between the closed state, which transited from the closed state and then back to the closed state; and oto is the transition state between the open state, which transited from the open state and then back to the open state.

To elucidate the association and dissociation mechanism of the seed base 5′-A and the target base 3′-U, we also calculated the RMSD of the two bases relative to their starting positions and the corresponding backbone torsion angles ζ. Figure 2A shows the time-dependent RMSD relative to the starting structure of the base pair of the seed base 5′-A and the target base 3′-U. Similar to that of the AU base pair in the absence of the PIWI/MID domain (Wang et al. 2016), the values of the RMSD distributed in a wide range (from 0 to 12 Å) but clearly centered on two regions, as shown in Figure 2B. In the first region, the RMSD fluctuated about 0.7 Å, indicating that the atoms of the seed base 5′-A and the target base 3′-U only vibrate around their initial positions and the base pair and base stacking were intact. In the other region, the RMSD was centered around 2 Å and it distributed in a wide range. The time-dependent RMSD of the seed base 5′-A and the target base 3′-U relative to the starting structures showed different distribution behavior, as shown in Figure 2C,D. The RMSD of the target base 3′-U relative to its starting structure was distributed in a wide range and the distributions were clearly centered on 0.7 and 3 Å regions, as shown in Figure 2D. However, the RMSD of the seed base 5′-A was almost only centered at 0.7 Å, as shown in Figure 2C, which denotes that the corresponding conformations of the seed base 5′-A vibrated only at its starting position.

We also calculated the backbone torsion angles ζ (see Figs. 3A, 4B) of the seed base 5′-A and target 3′-U. Similar to the case without the protein, the torsion angle ζ of the target base 3′-U was clearly centered on −75° and 50° regions (Fig. 3C), whereas the torsion angle ζ of the 5′-A was always in the region of −75° (Fig. 3B). The RMSD and the torsion angle ζ showed consistency (Figs. 4A,B): When RMSD was in the first region (0.7 Å), the torsion angle ζ was also in the first region (−75°); and when RMSD was in the second region (2 Å), the torsion angle ζ was also in the second region (50°). It has been found that the torsion angle ζ in the helix and coil state is just distributed about −75° and 50°, respectively (Hershkovitz et al. 2003). So according to the RMSD and the torsion angle, all the conformations of the seed base 5′-A and target 3′-U base pair are classified into three states as that without protein: association state (closed state), dissociation state (open state), and transition state. So even the 5′-A of the seed nucleotide was not paired with the target nucleotide 3′-U; it still kept its position as it paired, denoting a preorganization of the seed region.

### The reduced entropy penalty enhances the affinity of the seed-target interaction

**FIGURE 5.**
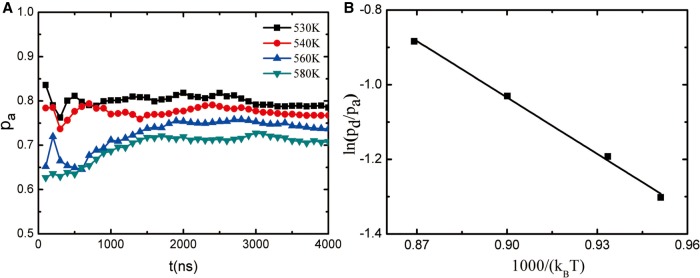
(*A*) The probability of the association state during the simulation time at the following simulation temperatures: 530, 540, 560, and 580 K. (*B*) The ln(*p*_*d*_/*p*_*a*_) at different simulation temperatures. Symbol: simulation results; line: linear fit.

At each simulation temperature, the probability of the association, dissociation, and transition states can be obtained as

$$p_{a}=\overset{N_{a}}{\underset{i=1}{\sum }}\tau_{i}^{a}/\tau ,\;p_{d}=\overset{N_{d}}{\underset{i=1}{\sum }}\tau_{i}^{d}/\tau ,\;p_{t}=\overset{N_{t}}{\underset{i=1}{\sum }}\tau_{i}^{t}/\tau ,$$

respectively, where *τ* is the total simulation time, and Na, Nd, and Nt are the total number of occurrences of the corresponding conformations that are in the association, dissociation, and transition states, respectively. $\tau_{i}^{a}$, $\tau_{i}^{d}$, and $\tau_{i}^{t}$ are the period in which the conformations are staying at the *i*th times in the association, dissociation, and transition states, respectively. Figure 5A showed the occupied probabilities of the association state during the simulation at all the simulation temperatures, in which each point is calculated at the time interval from initial to corresponding simulation time. It can be seen that for all the simulation temperatures, when the simulation time gets to 3600 nsec, the occupied probability of the association state reached a stable value, denoting the association-dissociation equilibration. In order to make sure the simulation reaches the association-dissociation switch equilibrium, each simulation temperature lasted 4000 nsec. The probabilities of the association, dissociation, and transition states at the simulation temperatures are listed in Table 1.

**TABLE 1.**
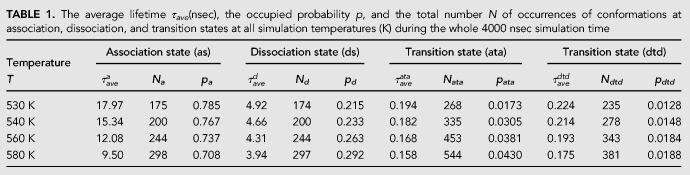
The average lifetime *τ*_*ave*_(nsec), the occupied probability *p*, and the total number *N* of occurrences of conformations at association, dissociation, and transition states at all simulation temperatures (K) during the whole 4000 nsec simulation time

Table 1 showed that the transition states only occupied negligible probabilities. So the conformations of the seed-target base pair were first treated as association and dissociation two-states. Therefore, the free energy difference Δ*G* for the association of the third seed nucleotide with the target can then be obtained through the equilibrate probabilities of the association and dissociation states:

(1) $$\Delta G=k_{B}Tln\left(\frac{p_{d}}{\;p_{a}}\right),$$

where *p*_*d*_ and *p*_*a*_ are the probability of the dissociation state and association state at temperature *T*, respectively; *k*_*B*_ is the Boltzmann constant.

As shown in Figure 5B, the ln(*p*_*d*_/*p*_*a*_) and the reciprocal of temperature (*1/T*) presented a linear relation, indicating the linear relationship of the free energy difference Δ*G* and the temperature *T*. As Δ*G* = Δ*H* − *T*Δ*S*, where Δ*H* and Δ*S* are the enthalpy and entropy changes for the transition between the association state and the dissociation states, respectively, the thermodynamic parameters of the seed-target AU base pair are found to be Δ*H* = −4.95 kcal/mol and Δ*S* = −6.78 eu, while they are Δ*H* = −7.3 kcal/mol and Δ*S* = −18.5 eu for the same terminal base pair without the PIWI/MID domain (Wang et al. 2016). Although the enthalpy change was decreasing in the presence of the protein, the free energy difference Δ*G* = Δ*H* − *T*Δ*S* between the association and dissociation state in the presence/absence of protein are −2.85 kcal/mol and −1.56 kcal/mol at *T* = 310 K, respectively. So the presence of the PIWI/MID domain enhances the interaction between the seed and target RNA through a reduced entropy penalty.

### The PIWI/MID domain of Argonaute protein reduces the barrier for association/dissociation of the seed and target base pair

**FIGURE 6.**
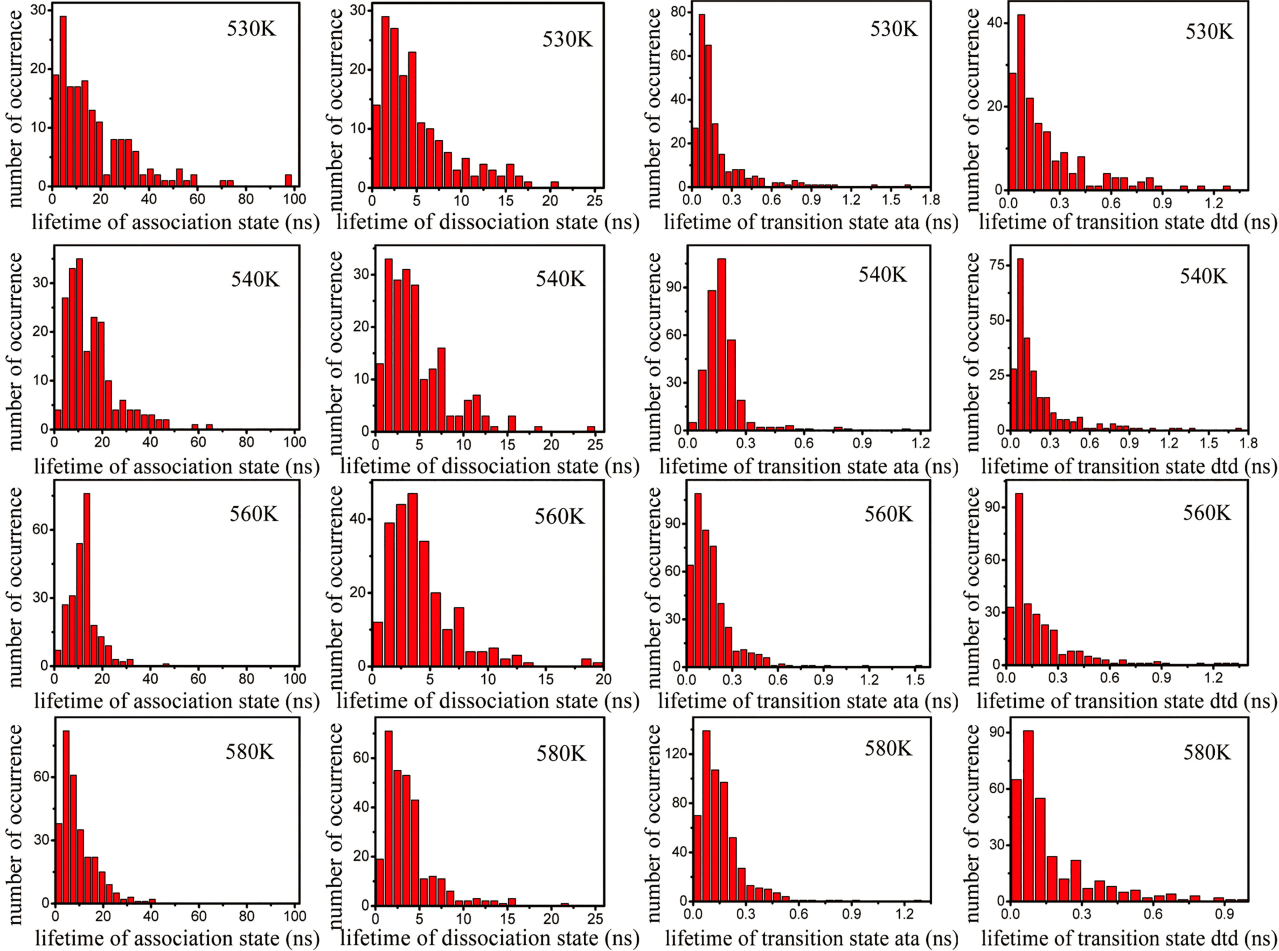
Histograms of the lifetime distribution of the association state, dissociation state, transition state (ata and dtd) at temperatures of 530, 540, 560, and 580 K.

**FIGURE 7.**
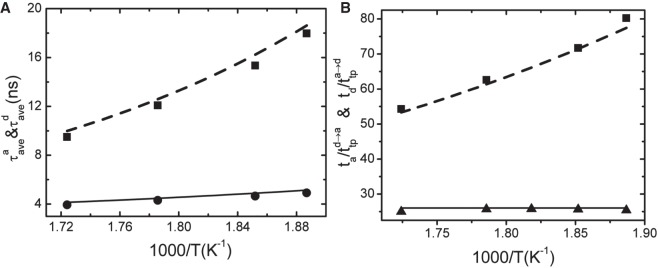
(*A*) The average lifetime of the association state (square) and the dissociation state (circle) at the simulation temperatures. Symbol: simulated results. Lines: fitted with Equation 2. (*B*) The ratios of $t_{a}/t_{tp}^{a\to d}$ (square) and $t_{d}/t_{tp}^{d\to a}$ (triangle). Symbol: simulated results. Lines: fitted via Equations 2 and 3.

The average lifetimes of the association state, dissociation state, and transition state can be obtained through $\tau_{ave}=\overset{N}{\underset{i=1}{\sum }}\tau_{i}/N$, where *τ*_*ave*_ is the average lifetime, *N* is the total number of occurrences of the 5′-A seed, and 3′-U target base pair in the association state or dissociation state or transition state, and *τ*_*i*_ is the *i*th dwelling period of the conformation in the corresponding state. Figure 6 showed the distribution of the lifetime of the association, dissociation, and transition states. The average lifetimes of the association and dissociation states at different temperatures are shown in Figure 7A. The average lifetime of the association state exhibited strong temperature dependence. However, that of the dissociation state was only weakly dependent on temperature, which was in agreement with the experimental finding that the folding and unfolding rates of RNA had different temperature-dependent behaviors (Craig et al. 1971).

For the seed-target base pair association-dissociation two-state transition, the association rate *k*_+_ and the dissociation rate *k*_−_ of the seed and target base pair can be obtained from the average lifetime of the dissociation and association states as $k_{+}=1/\tau_{ave}^{d}$, $k_{-}=1/\tau_{ave}^{a}$, where $\tau_{ave}^{d}$ and $\tau_{ave}^{a}$ are the average lifetimes of the dissociation state and association state, respectively.

The transition rates from the transition state to the association state *k*_*t*→*a*_ and to the dissociation state *k*_*t*→*d*_ can be acquired from the average lifetime of the transition states: $k_{t\to a}=1/\tau_{ave}^{ata}$ and $k_{t\to d}=1/\tau_{ave}^{dtd}$, where $\tau_{ave}^{ata}$ and $\tau_{ave}^{dtd}$ are the average lifetimes of the transition states ata and dtd, respectively.

As shown in Table 1, the average lifetime of the transition states was much shorter than the average lifetimes of the association and dissociation states, and it was also weakly dependent on temperature. Based on the transition-state theory (Berne et al. 1988; Hänggi et al. 1990; Chung and Eaton 2013), the average lifetime of the association state (*t*_*a*_) and the dissociation state (*t*_*d*_), the average transition path time from the dissociation state to the association state ($t_{tp}^{d\to a}$) and from the association state to the dissociation ($t_{tp}^{a\to d}$) are given by the following equations:

(2) $$\begin{array}{r} t_{a}=1/k_{-}=\frac{2\pi }{\beta D^{*}\omega^{*}\omega_{a}}\exp(\beta \Delta G_{a}),\\ t_{d}=1/k_{+}=\frac{2\pi }{\beta D^{*}\omega^{*}\omega_{d}}\exp(\beta \Delta G_{d}), \end{array}$$

(3) $$\begin{array}{rl} t_{tp}^{d\to a} & =\frac{1}{\beta D^{*}{\left(\omega^{*}\right)}^{2}}\ln(2e^{\gamma }\beta \Delta G_{d}),\\ t_{tp}^{a\to d} & =\frac{1}{\beta D^{*}{\left(\omega^{*}\right)}^{2}}\ln(2e^{\gamma }\beta \Delta G_{a}), \end{array}$$

where D* is the diffusion coefficient at the top of the free energy barrier; (ω*)^2^, (*ω*_*a*_)^2^, and (*ω*_*d*_)^2^ are the curvatures of the free energy surface at the top of the barrier, in the association and dissociation states, respectively; Δ*G*_*a*_ is the free energy barrier height from the association state to the dissociation state, Δ*G*_*d*_ is the free energy barrier height from the dissociation state to the association state, *β* = 1/*k*_*B*_ *T*, *k*_*B*_ is the Boltzmann constant; *T* is the temperature; and *γ* is Euler's constant.

It can be seen from Equations 2 and 3 that the ratios of $t_{a}/t_{tp}^{a\to d}$ and $t_{d}/t_{tp}^{d\to a}$ are independent of the diffusion coefficient D*, but only depend on the free energy barriers Δ*G*, ω*/*ω*_*a*_, and ω*/*ω*_*d*_. We found that $t_{d}/t_{tp}^{d\to a}$ was nearly temperature independent (Fig. 7B). Considering that the ratio ω*/*ω*_*d*_ is constant, as observed in the protein folding (Chung and Eaton 2013), then the free energy barrier of association for the seed 5′-A and target 3′-U base pair from the dissociation state should be directly proportional to temperature as Δ*G*_*d*_ ∝ *T*. The free energy difference of the association and dissociation states at temperature *T* was Δ*G* = Δ*G*_*a*_ − Δ*G*_*d*_ = Δ*H* − *T*Δ*S*. By fitting the two curves of $t_{a}/t_{tp}^{d\to a}$ and $t_{d}/t_{tp}^{a\to d}$ with the temperature, the free energy barrier from the association state to the dissociation state was Δ*G*_*a*_ = −4.95 kcal/mol, which coincides with the enthalpy change Δ*H* between the association state and dissociation state. The free energy barrier for the association of the seed 5′-A and target 3′-U was Δ*G*_*d*_ = *T*Δ*S*, where Δ*S* is exactly equal to the entropy difference between the dissociation and association states. So the barrier for the association of the seed miRNA and target mRNA was the reduction in entropy, Δ*S*, and the barrier for the dissociation of the seed miRNA and target mRNA was the increase in enthalpy, Δ*H*, by destroying the hydrogen bond and the base-stacking interactions. Therefore, the PIWI/MID domain would decrease the barrier for the seed-target association due to the reduced entropy penalty, which is conductive to binding the target quickly, and the decrease of the dissociation barrier could be helpful to rapidly dissociate from the wrong target.

### Conclusions

In conclusion, the thermodynamic and kinetic parameters of the third position in the seed region of the miRNA binding with the target were acquired by simulating the association–dissociation transition of this base pair in the presence of the PIWI/MID domain of the Argonaute protein. The results showed that in the presence of the protein, the entropy and enthalpy changes upon the seed base of the s(m)iRNA binding to the target were less than those in the absence of the protein. The binding affinity was increased due to the reduction of the entropy penalty, which resulted from the preorganization of the seed base into the A-helix form. The presence of the PIWI/MID domain would lower the association barrier resulting from the unfavorable entropy loss and the dissociation barrier coming from the disruption of hydrogen bonding and base-stacking interactions. These results indicate that the seed region is important for fast binding to the target, and would quickly dissociate when it was binding with the wrong target. As more structures of the RNA-protein complexes are given (Schirle et al. 2015; Sheu-Gruttadauria and MacRae 2018), the thermodynamic and kinetic properties of s(m)iRNA and target interactions over and outside the seed regions as well as the sequence and position dependence should be further studied in the future.

## MATERIALS AND METHODS

The Amber10 all-atom force field (Zgarbová et al. 2011) and Gromacs 4.5.6 (Pronk et al. 2013) were used to perform all the molecular dynamic simulations. The model system consists of an A-form duplex, which mimics nucleotides 2 to 6 over the seed region of guide RNA base-pairing with the messenger RNA, and the PIWI/MID domain of the Argonaute protein. The RNA sequence was r(AAUUU)·r(UUAAA). The starting structure of the RNA and protein complex (see Fig. 1) was obtained from the crystal structure (PDB ID: 4W5O) (Schirle et al. 2014). The protein contains N terminus, Linker 1, PAZ, Linker 2, MID, and PIWI domains, but the seed region was enveloped by Linker 2, PIWI, and MID domains. We retained the Linker 2 and PIWI/MID domains to build the system. The NaCl concentration was set to 0.5 M by adding Na^+^ and Cl^−^ ions (Joung and Cheatham 2008) at random initial positions. The TIP3P (Jorgensen et al. 1983; Mahoney and Jorgensen 2000) water model and the SETTLE algorithm was used to keep water molecules completely rigid (Miyamoto and Kollman 1992). The simulating box is set as a 9.9 × 9.1 × 9.0 nm triclinic box and periodic boundary conditions were used, ensuring three or four solvation layers in each direction. The final system had about 68,440 atoms. The simulations were performed at constant temperature and pressure by using velocity rescaling (Bussi et al. 2007) and the Parrinello–Rahman barostat algorithm (Parrinello and Rahman 1981), respectively. The Particle-Mesh Ewald method (Darden et al. 1993; Essmann et al. 1995) was used to treat the electrostatic interactions with a 10 Å direct space cutoff. Lennard-Jones interactions (Lennard-Jones 1931) were truncated at 10 Å. Bond lengths of the solute were constrained by the LINCS algorithm (Hess et al. 1997). The neighboring grid search method (Hess et al. 2008) was used and was updated every 10 steps. The equations of motion were integrated via the Verlet algorithm. The time step was 2 fsec and the coordinates were saved every 2 psec. The system was relaxed by energy minimization to equilibrium over 20 nsec at 290 K. The starting structure for the simulations at high temperatures was selected from the equilibrium structures. To obtain the thermodynamic and kinetic properties of the association and dissociation of the guide 5′-A and target 3′-U base pair in the presence of the PIWI/MID domain of Argonaute protein, all other atoms of the complex except those of the guide 5′-A and target 3′-U nucleotides were fixed with position restraint by using an additional force (force constant: 1000 kJ/mol nm^−2^). To effectively sample the conformation space of the associated and dissociated states, the simulation temperatures were 530, 540, 560, and 580 K; each simulation lasted 4000 nsec.

## References

[RNA069328WANC1] Bartel DP. 2004 MicroRNAs: genomics, biogenesis, mechanism, and function. Cell 116: 281–297. 10.1016/S0092-8674(04)00045-5 14744438

[RNA069328WANC2] Bartel DP. 2009 MicroRNAs: target recognition and regulatory functions. Cell 136: 215–233. 10.1016/j.cell.2009.01.002 19167326 PMC3794896

[RNA069328WANC3] Berne BJ, Borkovec M, Straub JE. 1988 Classical and modern methods in reaction rate theory. J Phys Chem 92: 3711–3725. 10.1021/j100324a007

[RNA069328WANC4] Bussi G, Donadio D, Parrinello M. 2007 Canonical sampling through velocity rescaling. J Chem Phys 126: 014101 10.1063/1.2408420 17212484

[RNA069328WANC5] Chandradoss SD, Schirle NT, Szczepaniak M, MacRae IJ, Joo C. 2015 A dynamic search process underlies microRNA targeting. Cell 162: 96–107. 10.1016/j.cell.2015.06.032 26140593 PMC4768356

[RNA069328WANC6] Chen J, Zhang W. 2012 Kinetic analysis of the effects of target structure on siRNA efficiency. J Chem Phys 137: 225102 10.1063/1.4769821 23249034

[RNA069328WANC7] Chung HS, Eaton WA. 2013 Single-molecule fluorescence probes dynamics of barrier crossing. Nature 502: 685–688. 10.1038/nature12649 24153185 PMC4009947

[RNA069328WANC8] Craig ME, Crothers DM, Doty P. 1971 Relaxation kinetics of dimer formation by self complementary oligonucleotides. J Mol Biol 62: 383–401. 10.1016/0022-2836(71)90434-7 5138338

[RNA069328WANC9] Darden T, York D, Pedersen L. 1993 Particle mesh Ewald: an *N*⋅log(*N*) method for Ewald sums in large systems. J Chem Phys 98: 10089–10092. 10.1063/1.464397

[RNA069328WANC10] Dieterich C, Stadler PF. 2013 Computational biology of RNA interactions. WIRES RNA 4: 107–120. 10.1002/wrna.1147 23139167

[RNA069328WANC11] Doench JG, Sharp PA. 2004 Specificity of microRNA target selection in translational repression. Genes Dev 18: 504–511. 10.1101/gad.1184404 15014042 PMC374233

[RNA069328WANC12] Dokholyan NV, Buldyrev SV, Stanley HE, Shakhnovich EI. 2000 Identifying the protein folding nucleus using molecular dynamics. J Mol Biol 296: 1183–1188. 10.1006/jmbi.1999.3534 10698625

[RNA069328WANC13] Enright AJ, John B, Gaul U, Tuschl T, Sander C, Marks DS. 2003 MicroRNA targets in *Drosophila*. Genome Biol 5: R1 10.1186/gb-2003-5-1-r1 14709173 PMC395733

[RNA069328WANC14] Essmann U, Perera L, Berkowitz ML, Darden T, Lee H, Pedersen LG. 1995 A smooth particle mesh Ewald method. J Chem Phys 103: 8577–8593. 10.1063/1.470117

[RNA069328WANC15] Gan HH, Gunsalus KC. 2013 Tertiary structure-based analysis of microRNA-target interactions. RNA 19: 539–551. 10.1261/rna.035691.112 23417009 PMC3677264

[RNA069328WANC16] Gong Z, Zhao Y, Chen C, Xiao Y. 2011 Role of ligand binding in structural organization of *add* A-riboswitch aptamer: a molecular dynamics simulation. J Biomol Struct Dyn 29: 403–416. 10.1080/07391102.2011.10507394 21875158

[RNA069328WANC17] Gorski SA, Vogel J, Doudna JA. 2017 RNA-based recognition and targeting: sowing the seeds of specificity. Nat Rev Mol Cell Biol 18: 215–228. 10.1038/nrm.2016.174 28196981

[RNA069328WANC18] Gregory RI, Chendrimada TP, Cooch N, Shiekhattar R. 2005 Human RISC couples microRNA biogenesis and posttranscriptional gene silencing. Cell 123: 631–640. 10.1016/j.cell.2005.10.022 16271387

[RNA069328WANC19] Hammond SM, Bernstein E, Beach D, Hannon GJ. 2000 An RNA-directed nuclease mediates post-transcriptional gene silencing in *Drosophila* cells. Nature 404: 293–296. 10.1038/35005107 10749213

[RNA069328WANC20] Hänggi P, Talkner P, Borkovec M. 1990 Reaction-rate theory: fifty years after Kramers. Rev Mod Phys 62: 251–341. 10.1103/RevModPhys.62.251

[RNA069328WANC21] Hershkovitz E, Tannenbaum E, Howerton SB, Sheth A, Tannenbaum A, Williams LD. 2003 Automated identification of RNA conformational motifs: theory and application to the HM LSU 23S rRNA. Nucleic Acids Res 31: 6249–6257. 10.1093/nar/gkg835 14576313 PMC275477

[RNA069328WANC22] Hess B, Bekker H, Berendsen HJ, Fraaije JG. 1997 LINCS: a linear constraint solver for molecular simulations. J Comput Chem 18: 1463–1472. 10.1002/(SICI)1096-987X(199709)18:12<1463::AID-JCC4>3.0.CO;2-H

[RNA069328WANC23] Hess B, Kutzner C, Van Der Spoel D, Lindahl E. 2008 GROMACS 4: algorithms for highly efficient, load-balanced, and scalable molecular simulation. J Chem Theory Comput 4: 435–447. 10.1021/ct700301q 26620784

[RNA069328WANC24] Hsu PW, Huang H-D, Hsu S-D, Lin L-Z, Tsou A-P, Tseng C-P, Stadler PF, Washietl S, Hofacker IL. 2006 miRNAMap: genomic maps of microRNA genes and their target genes in mammalian genomes. Nucleic Acids Res 34: D135–D139. 10.1093/nar/gkj135 16381831 PMC1347497

[RNA069328WANC25] Hutvagner G, Simard MJ. 2008 Argonaute proteins: key players in RNA silencing. Nat Rev Mol Cell Biol 9: 22–32. 10.1038/nrm2321 18073770

[RNA069328WANC26] Hutvágner G, Zamore PD. 2002 A microRNA in a multiple-turnover RNAi enzyme complex. Science 297: 2056–2060. 10.1126/science.1073827 12154197

[RNA069328WANC27] Jo MH, Shin S, Jung S-R, Kim E, Song J-J, Hohng S. 2015 Human Argonaute 2 has diverse reaction pathways on target RNAs. Mol Cell 59: 117–124. 10.1016/j.molcel.2015.04.027 26140367

[RNA069328WANC28] Jorgensen WL, Chandrasekhar J, Madura JD, Impey RW, Klein ML. 1983 Comparison of simple potential functions for simulating liquid water. J Chem Phys 79: 926–935. 10.1063/1.445869

[RNA069328WANC29] Joung IS, Cheatham TE. 2008 Determination of alkali and halide monovalent ion parameters for use in explicitly solvated biomolecular simulations. J Phys Chem B 112: 9020–9041. 10.1021/jp8001614 18593145 PMC2652252

[RNA069328WANC30] Kawamata T, Tomari Y. 2010 Making RISC. Trends Biochem Sci 35: 368–376. 10.1016/j.tibs.2010.03.009 20395147

[RNA069328WANC31] Khorshid M, Hausser J, Zavolan M, Van Nimwegen E. 2013 A biophysical miRNA-mRNA interaction model infers canonical and noncanonical targets. Nat Methods 10: 253–255. 10.1038/nmeth.2341 23334102

[RNA069328WANC32] Krek A, Grün D, Poy MN, Wolf R, Rosenberg L, Epstein EJ, MacMenamin P, Da Piedade I, Gunsalus KC, Stoffel M, 2005 Combinatorial microRNA target predictions. Nat Genet 37: 495–500. 10.1038/ng1536 15806104

[RNA069328WANC33] Krüger J, Rehmsmeier M. 2006 RNAhybrid: microRNA target prediction easy, fast and flexible. Nucleic Acids Res 34: W451–W454. 10.1093/nar/gkl243 16845047 PMC1538877

[RNA069328WANC34] Lennard-Jones JE. 1931 Cohesion. Proc Phys Soc 43: 461–482. 10.1088/0959-5309/43/5/301

[RNA069328WANC35] Lewis BP, Shih IH, Jones-Rhoades MW, Bartel DP, Burge CB. 2003 Prediction of mammalian microRNA targets. Cell 115: 787–798. 10.1016/S0092-8674(03)01018-3 14697198

[RNA069328WANC36] Lewis BP, Burge CB, Bartel DP. 2005 Conserved seed pairing, often flanked by adenosines, indicates that thousands of human genes are microRNA targets. Cell 120: 15–20. 10.1016/j.cell.2004.12.035 15652477

[RNA069328WANC37] Liu J, Carmell MA, Rivas FV, Marsden CG, Thomson JM, Song J-J, Hammond SM, Joshua-Tor L, Hannon GJ. 2004 Argonaute2 is the catalytic engine of mammalian RNAi. Science 305: 1437–1441. 10.1126/science.1102513 15284456

[RNA069328WANC38] Ma JB, Yuan YR, Meister G, Pei Y, Tuschl T, Patel DJ. 2005 Structural basis for 5′-end-specific recognition of guide RNA by the A. *fulgidus* Piwi protein. Nature 434: 666–670. 10.1038/nature03514 15800629 PMC4694588

[RNA069328WANC39] Mahoney MW, Jorgensen WL. 2000 A five-site model for liquid water and the reproduction of the density anomaly by rigid, nonpolarizable potential functions. J Chem Phys 112: 8910–8922. 10.1063/1.481505

[RNA069328WANC40] Maragkakis M, Reczko M, Simossis VA, Alexiou P, Papadopoulos GL, Dalamagas T, Giannopoulos G, Goumas G, Koukis E, Kourtis K, 2009 DIANA-microT web server: elucidating microRNA functions through target prediction. Nucleic Acids Res 37: W273–W276. 10.1093/nar/gkp292 19406924 PMC2703977

[RNA069328WANC41] Martinez J, Tuschl T. 2004 RISC is a 5′ phosphomonoester-producing RNA endonuclease. Genes Dev 18: 975–980. 10.1101/gad.1187904 15105377 PMC406288

[RNA069328WANC42] Martinez J, Patkaniowska A, Urlaub H, Lührmann R, Tuschl T. 2002 Single-stranded antisense siRNAs guide target RNA cleavage in RNAi. Cell 110: 563–574. 10.1016/S0092-8674(02)00908-X 12230974

[RNA069328WANC43] Miyamoto S, Kollman PA. 1992 SETTLE: an analytical version of the SHAKE and RATTLE algorithm for rigid water models. J Comput Chem 13: 952–962. 10.1002/jcc.540130805

[RNA069328WANC44] Nguyen Q, Khai K, Gomez YK, Bakhom M, Radcliffe A, La P, Rochelle D, Lee JW, Sorin EJ. 2017 Ensemble simulations: folding, unfolding and misfolding of a high-efficiency frameshifting RNA pseudoknot. Nucleic Acids Res 45: 4893–4904. 10.1093/nar/gkx088 28115636 PMC5416846

[RNA069328WANC45] Parker JS, Roe SM, Barford D. 2005 Structural insights into mRNA recognition from a PIWI domain-siRNA guide complex. Nature 434: 663–666. 10.1038/nature03462 15800628 PMC2938470

[RNA069328WANC46] Parker JS, Parizotto EA, Wang M, Roe SM, Barford D. 2009 Enhancement of the seed-target recognition step in RNA silencing by a PIWI/MID domain protein. Mol Cell 33: 204–214. 10.1016/j.molcel.2008.12.012 19187762 PMC2642989

[RNA069328WANC47] Parrinello M, Rahman A. 1981 Polymorphic transitions in single crystals: a new molecular dynamics method. J Appl Phys 52: 7182–7190. 10.1063/1.328693

[RNA069328WANC48] Pasquinelli AE. 2012 MicroRNAs and their targets: recognition, regulation and an emerging reciprocal relationship. Nat Rev Genet 13: 271–282. 10.1038/nrg3162 22411466

[RNA069328WANC49] Peters L, Meister G. 2007 Argonaute proteins: mediators of RNA silencing. Mol Cell 26: 611–623. 10.1016/j.molcel.2007.05.001 17560368

[RNA069328WANC50] Peterson SM, Thompson JA, Ufkin ML, Sathyanarayana P, Liaw L, Congdon CB. 2014 Common features of microRNA target prediction tools. Front Genet 5: 23 10.3389/fgene.2014.00023 24600468 PMC3927079

[RNA069328WANC51] Pronk S, Páll S, Schulz R, Larsson P, Bjelkmar P, Apostolov R, Shirts MR, Smith JC, Kasson PM, van der Spoel D, 2013 GROMACS 4.5: a high-throughput and highly parallel open source molecular simulation toolkit. Bioinformatics 29: 845–854. 10.1093/bioinformatics/btt055 23407358 PMC3605599

[RNA069328WANC52] Saito T, Sætrom P. 2010 MicroRNAs-targeting and target prediction. New Biotechnol 27: 243–249. 10.1016/j.nbt.2010.02.016 20219708

[RNA069328WANC53] Salomon WE, Jolly SM, Moore MJ, Zamore PD, Serebrov V. 2015 Single-molecule imaging reveals that argonaute reshapes the binding properties of its nucleic acid guides. Cell 162: 84–95. 10.1016/j.cell.2015.06.029 26140592 PMC4503223

[RNA069328WANC54] Schirle NT, Sheu-Gruttadauria J, MacRae IJ. 2014 Structural basis for microRNA targeting. Science 346: 608–613. 10.1126/science.1258040 25359968 PMC4313529

[RNA069328WANC55] Schirle NT, Sheu-Gruttadauria J, Chandradoss SD, Joo C, MacRae IJ. 2015 Water-mediated recognition of t1-adenosine anchors Argonaute2 to microRNA targets. eLife 4: e07646 10.7554/eLife.07646 PMC4606517 26359634

[RNA069328WANC56] Schuck J, Gursinsky T, Pantaleo V, Burgyán J, Behrens S-E. 2013 AGO/RISC-mediated antiviral RNA silencing in a plant in vitro system. Nucleic Acids Res 41: 5090–5103. 10.1093/nar/gkt193 23535144 PMC3643602

[RNA069328WANC57] Sheu-Gruttadauria J, MacRae IJ. 2018 Phase transitions in the assembly and function of human miRISC. Cell 173: 946–957. 10.1016/j.cell.2018.02.051 29576456 PMC5935535

[RNA069328WANC58] Song J-J, Smith SK, Hannon GJ, Joshua-Tor L. 2004 Crystal structure of Argonaute and its implications for RISC slicer activity. Science 305: 1434–1437. 10.1126/science.1102514 15284453

[RNA069328WANC59] Tang G. 2005 siRNA and miRNA: an insight into RISCs. Trends Biochem Sci 30: 106–114. 10.1016/j.tibs.2004.12.007 15691656

[RNA069328WANC60] van den Berg A, Mols J, Han J. 2008 RISC-target interaction: cleavage and translational suppression. Biochim Biophys Acta 1779: 668–677. 10.1016/j.bbagrm.2008.07.005 18692607 PMC2646505

[RNA069328WANC61] Várnai P, Canalia M, Leroy JL. 2004 Opening mechanism of G•T/U pairs in DNA and RNA duplexes: a combined study of imino proton exchange and molecular dynamics simulation. J Am Chem Soc 126: 14659–14667. 10.1021/ja0470721 15521786

[RNA069328WANC62] Vella MC, Reinert K, Slack FJ. 2004 Architecture of a validated microRNA::target interaction. Chem Biol 11: 1619–1623. 10.1016/j.chembiol.2004.09.010 15610845

[RNA069328WANC63] Wang Y, Juranek S, Li H, Sheng G, Tuschl T, Patel DJ. 2008a Structure of an argonaute silencing complex with a seed-containing guide DNA and target RNA duplex. Nature 456: 921–926. 10.1038/nature07666 19092929 PMC2765400

[RNA069328WANC64] Wang Y, Sheng G, Juranek S, Tuschl T, Patel DJ. 2008b Structure of the guide-strand-containing argonaute silencing complex. Nature 456: 209–213. 10.1038/nature07315 18754009 PMC4689319

[RNA069328WANC65] Wang Y, Li Y, Ma Z, Yang W, Ai C. 2010 Mechanism of microRNA-target interaction: molecular dynamics simulations and thermodynamics analysis. PLoS Comput Biol 6: e1000866 10.1371/journal.pcbi.1000866 20686687 PMC2912339

[RNA069328WANC66] Wang Y, Gong S, Wang Z, Zhang W. 2016 The thermodynamics and kinetics of a nucleotide base pair. J Chem Phys 144: 115101 10.1063/1.4944067 27004898

[RNA069328WANC67] Wang Y, Wang Z, Wang Y, Liu T, Zhang W. 2018 The nearest neighbor and next nearest neighbor effects on the thermodynamic and kinetic properties of RNA base pair. J Chem Phys 148: 045101 10.1063/1.5013282 29390847

[RNA069328WANC68] Wilson RC, Doudna JA. 2013 Molecular mechanisms of RNA interference. Annu Rev Biophys 42: 217–239. 10.1146/annurev-biophys-083012-130404 23654304 PMC5895182

[RNA069328WANC69] Zamore PD, Tuschl T, Sharp PA, Bartel DP. 2000 RNAi: double-stranded RNA directs the ATP-dependent cleavage of mRNA at 21 to 23 nucleotide intervals. Cell 101: 25–33. 10.1016/S0092-8674(00)80620-0 10778853

[RNA069328WANC70] Zgarbová M, Otyepka M, Šponer JI, Mládek AT, Banáš P, Cheatham TE III, Jurecka P. 2011 Refinement of the Cornell et al. nucleic acids force field based on reference quantum chemical calculations of glycosidic torsion profiles. J Chem Theory Comput 7: 2886–2902. 10.1021/ct200162x 21921995 PMC3171997

[RNA069328WANC71] Zhang J, Qin M, Wang W. 2006 Folding mechanism of β-hairpins studied by replica exchange molecular simulations. Proteins 62: 672–685. 10.1002/prot.20813 16362933

[RNA069328WANC72] Zhang J, Lin M, Chen R, Wang W, Liang J. 2008 Discrete state model and accurate estimation of loop entropy of RNA secondary structures. J Chem Phys 128: 03B624 10.1063/1.2895050 PMC2494904 18376982

[RNA069328WANC73] Zhang Y, Zhang J, Wang W. 2011 Atomistic analysis of pseudoknotted RNA unfolding. J Am Chem Soc 133: 6882–6885. 10.1021/ja1109425 21500824

